# Involvement of a Microplusin-like Gene (HlonML-1) in the Olfactory Chemosensation of *Haemophysalis longicornis:* Expression, RNA Silencing, and Behavioral Implications

**DOI:** 10.3390/microorganisms12112269

**Published:** 2024-11-08

**Authors:** Mebrahtu Berhe Gebremedhin, Zhengmao Xu, Ceyan Kuang, Mohsin Nawaz, Nana Wei, Jie Cao, Yongzhi Zhou, Houshuang Zhang, Jinlin Zhou

**Affiliations:** Key Laboratory of Animal Parasitology of the Ministry of Agriculture, Shanghai Veterinary Research Institute, Chinese Academy of Agricultural Sciences, Shanghai 200241, China1987wnn@163.com (N.W.);

**Keywords:** chemosensation, gene expression, functional analysis, *Haemophysalis longicornis*, identification, microplusin-like gene

## Abstract

The study of tick olfaction is relatively new compared to that of insects, and the molecular mechanisms involved remain poorly understood. Despite several potential chemosensory genes identified in multiple tick species, these are yet to be validated through independent functional experiments. In this research, we cloned and analyzed a microplusin-like gene, HlonML-1, and investigated its role in the chemosensory activities of *H. longicornis*. The results showed that this gene’s amino acid sequences lack histidine residues essential for antimicrobial activity, and it is evolutionarily linked to putative chemosensory microplusins in ticks. Gene expression analyses indicated that HlonML-1 was significantly more abundant in ticks exposed to potential attractants and in the forelegs of *H. longicornis* than in non-exposed ticks and the hindlegs, respectively. Tick forelegs support the Haller’s organ, which is a sensory structure mostly specialized for chemosensation. Furthermore, Y-tube olfactometer assays indicated that silencing HlonML-1 significantly impaired adult ticks’ ability to detect selected odors, while their gustatory-related behavior remained unaffected compared to the control groups. Given its unique sequences, relative abundance in chemosensory tissues, and impact on odor detection, HlonML-1 is likely involved in the olfactory chemosensation of *H. longicornis*. Future research validating putative chemosensory microplusins in the genomes of various tick species may enhance our understanding of their olfactory functions in tick and lead to the identification of new molecular targets for developing tick repellents.

## 1. Introduction

Ticks are ectoparasites that act as vectors for pathogens affecting animals and humans [1,2], requiring blood meals to grow and reproduce. They must recognize their hosts to feed [3], relying primarily on olfaction [4,5], since they have rudimentary eyes [6] and no hearing organs [7]. Chemosensation in ticks is mostly attributed to a unique chemosensory structure, the “Haller’s organ”, on their forelegs [8,9,10], which is thought to play a crucial role in their host-seeking behavior [5,11]. In addition to scanning electron microscopy-based structural investigations, behavioral, electrophysiological, and other relevant studies have also highlighted the importance of this organ in detecting attractants [12] and its potential as a target for developing new tick repellents [5,13]. Understanding tick interactions with their environment [14] and the relevant chemosensory receptors [15] is essential for creating effective management strategies.

Arthropod chemoreception, primarily studied in the fruit fly *Drosophila melanogaster*, involves three multigene families of chemosensory receptors—gustatory receptors (GRs) [16], odorant receptors (ORs) [17], and ionotropic receptors (IRs) that are part of the ancient ionotropic glutamate receptor family [18]. Additionally, two families of soluble binding proteins, namely chemosensory proteins (CSPs) and insect-type odorant-binding proteins (OBPs), facilitate the transport of ligands to these receptors [19,20]. Unfortunately, unlike that of insects, olfaction in ticks is a relatively recent topic of research interest, and little is known about its molecular basis [5]. Recently, tick foreleg-based transcriptome and proteomic studies have revealed several candidate chemoreceptors, which are mostly based on whole tick-genome-based sequence data from different tick species, as reviewed in our previous work [21]. Members of the IR and GR families dominate, while the involvement of candidate G-protein coupled receptors (GPCRs) is yet uncertain. The main chemoreceptors in insects, the ORs, are yet to be reported in ticks. Additionally, genes encoding small, soluble potential binding proteins including the Niemann pick type-c 2 (NPC2s) and microplusin-like (MLs) proteins, have recently been shown to play a role in tick chemosensation, mostly via olfaction.

As scant data are available to support the presence of the two classes of insect-like soluble proteins (CSPs and OBPs) to date [19,22], instead, the authors suggest that this new class of soluble proteins, NPC2s [20,23,24] and MLs [22,25,26], might replace their role in ticks, binding and transporting semiochemicals from the environment to chemoreceptors. This drives the hypothesis that ticks might have evolved to recruit new chemosensory-related molecules in response to adopting a new chemical ecology unlike insects [5,21,25]. Since this research area is still emerging, further investigations focused on identifying and deorphanizing [15] key chemoreceptor genes are crucial for a thorough understanding of the molecular mechanisms underlying chemosensation in ticks, to which existing high-quality tick genomes may also contribute.

Microplusin, a cysteine-rich antimicrobial peptide (AMP) with histidine-rich regions at the N and C termini, was primarily characterized in the cattle tick *R. microplus* [27]. However, interestingly, several microplusins in *Ixodes scapularis* (*I. scapularis)* and *Ixodes ricinus (I. ricinus*) lack their N-terminal histidine-rich domain [28], which is important for their bacteriostatic effect via chelating copper ions. This likely makes them lack the typical feature of being innate defense molecules in ticks [29]. In addition to the sequence-based evidence, which casts doubt on the antimicrobial role of these microplusins, a foreleg-based comparative analysis identified several microplusin-like genes (MLs) differentially expressed in the forelegs of *I. scapularis* [25] and *Amblyomma americanum* (*A. americanum*) [22] and proposed that these are involved in tick chemosensory-related activities.

This research aimed to identify a chemosensory-related microplusin-like gene by examining three target genes from the transcriptome of the parthenogenic *Haemophysalis longicornis*, which is a tick species widely found in eastern Asia that poses significant public health risks, even in developed countries [26,30]. The researchers utilized sequence analysis to screen for a potential gene, corroborating the results with quantitative real-time PCR to assess the target gene’s expression patterns in chemosensory tissues and beyond. Functional analyses were also conducted using gene-silencing techniques alongside tick chemosensory-related behavioral bioassays to investigate the gene’s influence on odorant recognition and attachment rates to rabbit ears. Despite RNA interference (RNAi) being extensively applied in insects, particularly mosquitoes, to explore a particular gene’s chemosensory function [31,32,33], this study represents the first instance of using RNAi to evaluate chemosensory-related gene functions in ticks—a technique that has been employed for other gene types for over twenty years [34]. The authors believe this work may inspire further investigation into similar genes.

## 2. Materials and Methods

### 2.1. Ticks and Semiochemicals

All stages of *Haemophysalis longicornis* (parthenogenetic strain) ticks were maintained in our laboratory at the Shanghai Veterinary Research Institute (SHVRI) of the Chinese Academy of Agricultural Sciences (CAAS) in Shanghai, China. The ticks were fed on the ears of New Zealand white rabbits and kept in a dark incubator at 25 °C with 95% humidity, as described by Zhou et al. [35]. Fresh ticks, about two weeks post-molting, were used in our experimental tests.

Ammonia solution (NH_4_OH) (25~28%) was purchased from Sinopharm Chemical Reagent Co., Ltd. (Shanghai, China), while *N*,*N*-Diethyl-3-methylbenzamide (DEET) (99%) was obtained from Sigma-Aldrich (St. Louis, MO, USA). Tick exposure to carbon dioxide (CO_2_) gas was conducted using an adjustable Thermo Scientific Hera Cell 240i CO_2_ incubator (Thermo Fisher Scientific Inc., Waltham, MA, USA) set to 5% CO_2_. We evaluated the olfactory-related behavioral responses of *H. longicornis* ticks using a Y-tube apparatus [36], considering commonly used tick repellent (20% DEET) and attractant (5% NH_4_OH) [37,38], which were also verified in our preliminary dose–response tests.

### 2.2. Tissue Collection, RNA Extraction, and cDNA Synthesis

Tissue samples were collected from *H. longicornis* ticks at various developmental stages (larvae, nymphs, and adults) and feeding statuses (unfed and fed). The hard tissues (mouth parts, forelegs, and hindlegs) of unfed adult ticks were removed directly using scalpel blades. Hemolymph was collected by cutting the coxae of the ticks at the distal joint. For the visceral organs (fat body, salivary glands, ovary, and midgut), a small incision was made along the dorsolateral margin of partially engorged adult ticks, and the dorsal surface was carefully removed. The coelom was then washed with cooled phosphate-buffered saline (PBS), and the cleaned target organs were collected under a light microscope. These samples were immediately pooled into 2 mL RNase-free tubes and placed on pelleted dry ice. Five unfed adult ticks and thirty nymph ticks were immediately collected after 30 min of exposure to selected odors or following RNA interference; these samples were stored at −80 °C for future use [35].

The live ticks or tissue samples were snap-frozen in liquid nitrogen; then, they were crushed and homogenized using a freeze-miller MB-48LD tissue grinding machine with beads (Meibi Instrument Co., Ltd., Hangzhou, China) set to −20 °C. Approximately 100 mg of tissue was ground with 1 mL of TRIzol reagent (Invitrogen, Carlsbad, CA, USA) for total RNA extraction or with PBS for subsequent Western blot analysis. Following centrifugation at 12,000 rpm for 10–15 min at +4 °C, and 5–10 min incubation at room temperature, the tissue samples were treated with 0.2 mL of chloroform and then 0.5 mL of isopropanol. The RNA pellets were dissolved in 1 mL of 75% pre-cooled ethanol and collected in 20–50 μL of double-distilled water. The quality of the extracted RNA was assessed using a Nanodrop2000 spectrophotometer from NanoDrop Technologies (Thermo Fisher Scientific, Waltham, MA, USA). For cDNA synthesis, the PrimeScript™ II First Strand cDNA Synthesis Kit (Takara Biotechnology Co. Ltd., Dalian, China) and HiScript R III RT SuperMix for qPCR (+gDNA wiper) kit (Vazyme Biotech, Nanjing, China) were utilized. All the tissue grinding, RNA extraction, and cDNA synthesis steps were performed according to the respective manufacturers’ protocols.

### 2.3. Gene Cloning and Sequence Analysis

Three transcript sequences potentially encoding microplusin-like genes were targeted in this study. One sequence designated HlonML-1 was derived from a transcript expressed in the fat body of parthenogenetic *H. longicornis* [GenBank: HY962019.1]. The other two sequences, Unigene495 (HlonML-2) and Unigene570 (HlonML-3), were obtained from an annotated cDNA library of the transcriptome of unfed nymphs exposed to CO_2_ gas, which was conducted in our laboratory at SHVRI. We followed the naming style established by Cui et al. [23] for the olfactory-related genes identified in *H. longicornis.* Specific primers (see Supplementary Table S2) for HlonML-1 were designed based on its open reading frame (ORF), and a 297 bp band was amplified using the Takara PrimerSTAR Max DNA Polymerase kit (Takara Biotechnology Co., Ltd., Dalian, China), following the manufacturer’s protocol. A no-template control was included to detect potential contamination. The pMD-19T vector was used to ligate the amplicons containing the complete ORF sequence, and the resulting clones were sequenced. Selected clones containing the gene of interest were subsequently used as templates for downstream experiments, including double-strand RNA (dsRNA) synthesis and subcloning into expression vectors (pET-32a and pGEX-6T-1).

Signal peptide sequences of the matured proteins (HlonML-1, 2, and 3) were predicted from the deduced amino acid sequences of the ORFs, using the online server SignalP-6.0 (https://services.healthtech.dtu.dk/services/SignalP-6.0/), accessed on 5 January 2023 [39]. The theoretical isoelectric point (pI) and molecular weight for HlonML-1 were predicted using the expassy tool (https://web.expasy.org/cgi-bin/compute_pi/pi_tool.cgi), also accessed on 5 January 2023. A basic local alignment search tool (BLAST) analysis of the deduced amino acid sequences was performed to identify homologous proteins. The amino acid sequences of the target genes were aligned with that of microplusin AMP precursor protein using Genetyx 6 (Genetyx, Tokyo, Japan), evaluating the shared sequence features related to antimicrobial activities. For phylogenetic analysis, the amino acid sequences of the target genes were compared with those of related putative chemosensory microplusins (MLs) identified in ticks [22,25]. These sequences were aligned using the Muscle algorithm and analyzed using the maximum likelihood method with default settings in MEGA X [40]. The amino acid sequences of the targeted ORFs used for multiple sequence alignment and evolutionary analysis are presented in Supplementary Table S1.

### 2.4. Recombinant Protein Expression, and Polyclonal Antibody Preparation

The HlonML-1 recombinant protein generates a polyclonal antibody in mice for subsequent target protein detection. Specific primers (see Supplementary Table S2) were designed to amplify the ORF sequence of the target gene from the template ligated into the pMD-19T vector. The purified and enzyme-digested PCR fragments were then subcloned into either the pET-32a or pGEX-6T-1 vectors (Invitrogen, Carlsbad, CA, USA) using the In-Fusion HD Cloning Kit (Takara Clontech, Mountain View, CA, USA). Recombinant plasmids obtained from positive clones were transformed into BL21 (DE3) *Escherichia coli* competent cells (TIANGEN, Beijing, China). The strains were cultivated at 37 °C until the optimal density at 600 nm (OD600) reached 0.8. Protein expression was then enhanced at 37 °C for 4 h with 1 mM isopropyl-d-1-thiogalactopyranoside (IPTG). The resulting recombinant proteins, which contained His tags or glutathione-S-transferase (GST) tags, were purified by affinity chromatography using Ni–NTA His resin (Thermo Fisher Scientific, Waltham, MA, USA) or glutathione resin via gravity flow, respectively.

Antibodies against the HlonML-1 protein were raised in seven BALB/C mice maintained in a specific-pathogen-free (SPF) animal area through intraperitoneal immunization. The mice were inoculated three times at 15-day intervals. Specifically, 100 μg of the purified recombinant protein (Gst-HlonML-1) was emulsified in PBS with an equal volume of Freund’s complete adjuvant (Sigma-Aldrich, St. Louis, MO, USA) and administered. This was followed by two booster doses of 50 μg of the purified protein in PBS, which was emulsified with an equal volume of Freund’s incomplete adjuvant (Sigma-Aldrich, St. Louis, MO, USA). Blood samples were collected seven days after the third injection, and the serum was stored at −20 °C until needed.

### 2.5. Western Blotting

Total protein was extracted from various tissues including the forelegs of *H. longicornis*, and Western blot analysis was conducted to detect the target protein [41]. Briefly, 2 to 3 µg of purified recombinant HlonML-1 protein and 20 to 30 µg of tissue extract protein were applied to sodium dodecyl sulfate-polyacrylamide gel electrophoresis (SDS-PAGE; 12%, Gene script, Nanjing, China). The gel-separated protein was stained with Coomassie blue and visualized using a gel-doc molecular imager (Bio-Rad, Hercules, CA, USA). The SDS-PAGE separated proteins were transferred onto polyvinylidene difluoride (PVDF) membranes for immune blotting. The PVDF membrane was incubated overnight at 4 °C with primary antibodies against His-Tag (66005-1-Ig, Protein Tech., Chicago, IL, USA) or Gst-HlonML-1. Then, they were incubated with a secondary antibody: goat anti-mouse conjugated with horseradish peroxidase (HRP, 31430; 1:5000, Thermo Fisher Scientific, Waltham, MA, USA) for 2 h at room temperature. Serum from anti-Gst-HlonML-1 was used to target HlonML-1 in tissue protein extracts. The signal was detected using an Enhanced Chemiluminescent Substrate Reagent Kit (NCM Biotech, Suzhou, China), and images were captured with the ChemiDoc Touch imaging system (Bio-Rad, Hercules, CA, USA).

### 2.6. Gene Expression Analysis (RT-qPCR)

The expression patterns of HlonML-1 were examined across different developmental stages and feeding statuses, as well as in chemosensory versus (Vs) non-chemosensory tissues, and in *H. longicornis* exposed to odors vs. those not exposed. cDNA samples (1–2 μL) synthesized from RNA extracts of these tissues were added to a reaction mix (18–19 μL) prepared using ChamQ Universal SYBR qPCR Master Mix (Q711, Vazyme Biotech, Nanjing, China), which included the designed primers (see Supplementary Table S2). The cDNA was analyzed using a QuantStudio™ 5 Real-Time polymerase chain reaction (PCR) System (Applied Biosystems™, Waltham, MA, USA) under the following reaction conditions: 95 °C for 30 s; 40 cycles of 95 °C for 10 s and 60 °C for 30 s; followed by 95 °C for 15 s, 60 °C for 60 s, and a final step at 95 °C for 15 s. Data were normalized against the expression of the stable reference gene elongation factor-1α (ELF1α) [GenBank: AB836665], serving as an internal control. A negative control (NC) sample without the cDNA template was included to detect potential contamination from extraneous nucleic acids. Each reaction was executed in triplicate, and the expression level of HlonML-1 in samples with and without the control group was calculated using the 2^−∆∆Ct^ [23] and 2^−∆Ct^ [42] analytical methods, respectively.

### 2.7. RNA Interference Alongside Ticks’ Chemosensory-Related Behavioral Assays

An RNA interference (RNAi) experiment was designed to target the HlonML-1 gene with dsRNA synthesis and tick microinjection performed by modifying the methods described in previous studies [43,44]. The HlonML-1-specific primers containing T7 promoter sequences (see Supplementary Table S2) were designed for PCR amplification using the pMD19-T template. The dsRNA was synthesized from the purified PCR products using the T7 RiboMAX™ RNAi System (P1700, Promega, Madison, WI, USA), following the manufacturer’s recommendations. Unfed ticks were micro-injected with approximately 0.5 μL of dsRNA (~1 μg/μL) for adults and 9.2 nL of dsRNA (1.5 μg/μL) for nymph [45], using a Nanoject II system (Drummond Scientific, Broomall, PA, USA). The injections were administered at the lower right quadrant of the ventral surface of their exoskeleton [43]. Control ticks were injected with unrelated dsRNA targeting luciferase (dsLuci) [44], and non-injected (blank) ticks were also included as a test control. The efficiency of gene knockdown was evaluated using RT-qPCR [46]. dsRNA-injected ticks were kept in an incubator for at least 24 h at 25 °C and 95% relative humidity (RH). Subsequently, biological parameters were analyzed at room temperature in a controlled environment within our tick experimental room. The chemosensory-related behavioral responses of both treatment and control groups were assessed in parallel, focusing on the olfactory response to selected odorants and gustatory responses measured by attachment rates to rabbit ears.

In the Y-tube olfactometer assay, dsRNA-treated (dsHlonML-1 & dsLuci) and untreated groups of unfed adult or nymph *H. longicornis* (20 ticks per group) were placed into a 15 mL plastic container attached to a Y-tube at the top, as described by Zhou et al. [36]. A piece of cellulose filter paper (1 cm^2^) treated with 20 µL of either the control (solvent: double-distilled water or pure ethanol) or odorant solutions (5% NH_4_OH and 20% DEET) was applied to each tip of the Y-tube ports and then secured with a clean cloth mesh and rubber. All ticks were acclimated to the experimental setting for about 30 min before being transferred into the olfactometer. Dead or inactive ticks were immediately replaced before testing while activating them with human breath [43]. The olfactometer was positioned vertically for nymphs and horizontally for adults [38]. Ticks were recorded as positive responders only if they moved at least 2.5 cm into one of the arms of the Y-tube. Each bioassay lasted 5 min [47] and was replicated thrice with each tick only tested once. Attachment rates were recorded 24 h after the ticks were released to feed on shaved rabbit ears enclosed within an ear bag [41], assessing their gustatory-related behavioral responses, as potential kairomones are expected on the host’s skin. The experiment included dsRNA-treated groups for HlonML-1- (treatment) and luciferase (control) as well as untreated (blank) groups of unfed adult *H. longicornis.* The data analysis did not consider ticks that died during the experiment. Twenty ticks were tested per group per rabbit ear. Each test group was triplicated.

### 2.8. Ethical Consent

Ticks of *H. longicornis* were maintained on New Zealand white rabbits (JSJ Laboratory Animal Co., Ltd., Shanghai, China) by the Shanghai Veterinary Research Institute (SHVRI), Chinese Academy of Agricultural Sciences (CAAS). BALB/c mice (SPF Biotechnology Co., Ltd., Beijing, China) were used to prepare polyclonal antibodies. All experimental procedures involving animals adhered to the approved protocols of the Animal Care and Use Committee at SHVRI, CAAS.

### 2.9. Statistical Analysis

Statistical data analyses were performed using Prism 9.0 (GraphPad Software, San Diego, CA, USA). Results are presented as mean ± standard error of the mean (SEM) from triplicate measures [48]. Student’s *t*-test and ANOVA were used to identify significant differences (*p* < 0.05) between group means with significance levels indicated as follows: ns (not significant), * *p* < 0.05, ** *p* < 0.01, *** *p* < 0.001. In the Y-tube olfactometer assays, tests in which less than 35% of ticks responded and made a choice were excluded [49]. Additionally, ticks that did not choose either side of the Y-tube or were dead were excluded. The data were transformed and adjusted before analysis.

## 3. Results

### 3.1. The Putative Chemosensory-Related Microplusin-like Gene: Sequence Analysis

Microplusin-like genes (HlonML-1, -2, and -3) were identified from the transcriptome of a parthenogenic *H. longicornis.* The transcript sequences of these target genes are 473, 495, and 570 bp in length, containing open reading frames (ORFs) of 297, 330, and 324 bp that encode 98, 109, and 107 amino acid (aa) residues, respectively (see Supplementary Table S1). According to the basic local alignment search tool (BLASTp) analysis, HlonML-2 and -3 share significant sequence similarity with antimicrobial peptide (AMP) microplusin, showing 67.89% identity (E-value: 2 × 10^−44^) with the sequence [accession: XP_037559719.1] from *Dermacentor silvarum*. Additionally, they also exhibit 64.44% (E-value: 4 × 10^−31^) and 50.00% (E-value: 1 × 10^−22^) identity with the precursor AMP microplusin [accession: XP_037269734.1], which was primarily identified from *R. microplus*. This suggests a potential antimicrobial role for these two candidate genes. In contrast, the third candidate gene (HlonML-1) resembles multiple uncharacterized and hypothetical small secreted proteins in ticks, including those similar to microplusin (69.39%; E-value: 2 × 10^−42^) [accession: GenBank: JAP81958.1] and microplusin preprotein (40.21%; E-value: 2 × 10^−29^) [accession: EEC05278.1] from *Rhipicephalus appendiculatus* and *I. scapularis*, respectively. This indicates that HlonML-1 may diverge from the antimicrobial peptide, microplusin, while sharing 60.26% (E-value: 2 × 10^−36^) sequence identity with a putative chemosensory microplusin-like gene (ML3: A. Amer) from *Amblyomma americanum* [22].

**Figure 1:**
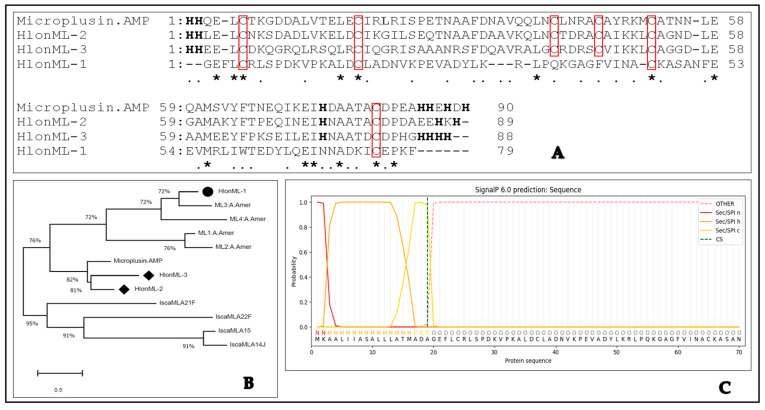
Deduced amino acid sequences analysis of candidate microplusin-like genes (MLs) in *Haemophysalis longicornis*. Target MLs were aligned with the precursor antimicrobial peptide, microplusin (XP_037269734.1), to highlight the number and positions of shared cysteine (boxed) and histidine (boldly highlighted) residues. This multiple protein sequence analysis was computed using Genetyx.6. Asterisks (*) denote those of highly conserved residues, while dots (.) indicate conservation between groups of weakly similar ones (**A**). Molecular evolutionary relationships analysis of the target MLs (boldly marked with bullets) were compared with their relatives in *A. Americanum* (A. Amer) [22], *I. scapularis* (Isca) [25], and *R. microplus* (Microplusin AMR) [accession: XP_037269734.1]. Bootstrap trees were inferred from 500 replicates, using a maximum-likelihood method in the MEGA X: Software [40]. (**B**). HlonML-1 Signal Peptide (Sec/SPI) sequences predicted with a likelihood of 0.9998, at a cleavage site between pos 19 and 20, and a probability of 0.9472. The signal peptide sequence’s n-terminal (N), hydrophobic (H), and c-terminal (C) regions were also indicated (**C**).

Multiple protein sequence analyses revealed that HlonML-2 and -3 share six conserved cysteines with the antimicrobial peptide (AMP) microplusin, while HlonML-1 shares four cysteines. Additionally, HlonML-2 and -3 retain similarities in the number and positions of histidine residues compared to AMP microplusins, which are absent in HlonML-1 (Figure 1A). This suggests that HlonML-1 has significantly diverged in sequence similarity and patterns from the precursor AMP microplusin, raising the possibility of a new functional role. According to the evolutionary relationship analysis (Figure 1B), HlonML-1 is related to putative chemosensory microplusin-like genes identified in *A. Americanum*. In contrast, HlonML-2 and -3 appear to be evolutionarily related to microplusin AMP, indicating that they may also play a similar antimicrobial role in *H. longicornis,* unlike HlonML-1. Their sequences appear highly conserved in ticks but show variability within the broader arthropod lineage. Bioinformatic analysis predicts that the matured proteins of these ORFs share a signal peptide sequence in their N-terminal regions, as illustrated for HlonML-1 in Figure 1C, although no significant conserved domains were detected. The ORF sequence of HlonML-1 predicts a theoretical isoelectric point (pI) of 5.07, and a molecular weight (Mw) of 10.8 kDa, while its potential matured protein is expected to have a pI of 5.04 and an Mw of 8.9 kDa.

### 3.2. Detection of HlonML-1 Gene and Its Recombinant Protein in H. longicornis

**Figure 2:**
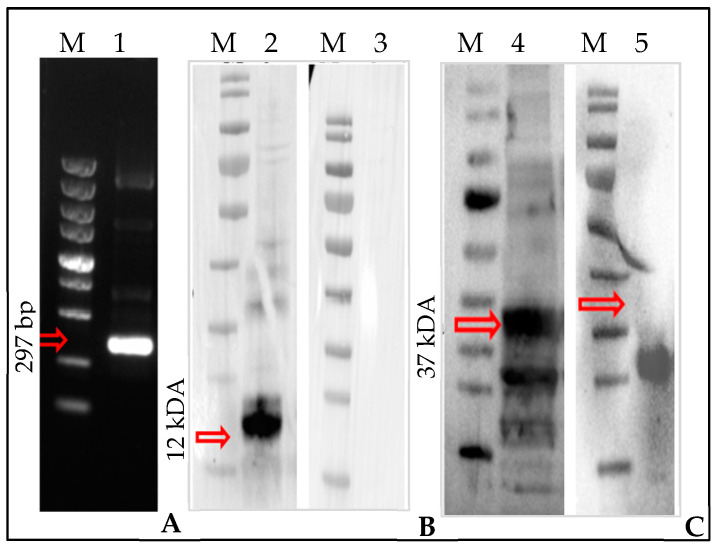
Detection of HlonML-1 in *H. longicornis*. Agarose gel electrophoresis: (M) molecular marker 100–2000 bp, (1) 297 base pairs (bp) long band (HlonML-1) (**A**). Western blot: (M) protein marker 10–170 kDa (2) 12 kDa His-HlonML-1 purified recombinant protein (3) control (no anti-his-tag) (**B**). Western blot: (M) protein marker 10–170 kDa, (4) 37 kDa Gst-HlonML-1 recombinant protein (5) control (mouse sera) (**C**).

Unlike the other two candidate genes, the bioinformatic evidence suggests a putative chemosensory role of HlonML-1. Consequently, we cloned and characterized this target gene in *H. longicornis*. HlonML-1 was PCR amplified, and a band of 279 bp was detected by agarose gel electrophoresis (Figure 2A). Furthermore, the HlonML-1 protein was recombinantly expressed and detected using Western blotting; an HIS-tagged purified recombinant protein of about 12 kDa was initially detected with an anti-HIS-tag monoclonal antibody (Figure 2B). However, considering its small size, this finding was further corroborated by identifying a 37 kDa GST-tagged recombinant protein (Figure 2C) in whole-body tissue extracts of adult *H. longicornis*, using anti-Gst-HlonML-1 serum prepared from a mouse. Unfortunately, this mouse-generated anti-Gst-HlonML-1 serum failed to detect the native HlonML-1 protein in the chemosensory tissue extracts (forelegs and mouth part) of *H. longicornis*. Therefore, we focused on further analyzing the function of the target gene.

### 3.3. HlonML-1 Gene Expression Patterns and Its Changes in Tick Olfactory Behavior

**Figure 3:**
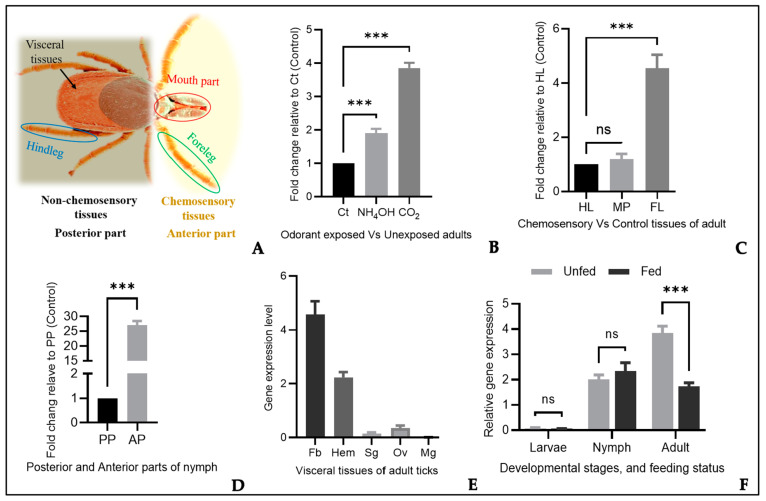
Gene expression patterns of HlonML-1 in *H. longicornis*. The chemosensory-related and non-chemosensory tissue parts of *H. longicornis* are considered in this study (**A**). Relative gene expression levels in adult ticks exposed to ammonia solution (NH_4_OH) and carbon dioxide (CO_2_) gas versus unexposed (control/Ct) ones (**B**). Relative gene expression levels in chemosensory tissues (foreleg/FL and mouth part/MP) versus non-chemosensory tissue (hindleg/HL) of the adult ticks (**C**). Relative gene expression levels in the anterior part/AP (including the chemosensory tissues) versus posterior parts/PP (control) of nymphs (**D**). The gene expression levels in visceral tissues; fat body (Fb), hemolymph (Hem), salivary gland (Sg), ovary (Ov), and midgut (Mg)) of a partially engorged (four days fed) adult tick (**E**). Relative gene expression levels in feed versus unfed ticks at different developmental stages (larvae, nymph, adult) (**F**). Asterisks denote levels of statistically significant differences (*** *p* < 0.001) and ns stands for no significant difference).

The chemosensory and non-chemosensory tissue parts of *H. longicornis* considered for this study are illustrated in the figure below (Figure 3A) and were used to determine the relative expression of the target gene. The forelegs of the ticks contain Haller’s organ, a unique chemosensory structure primarily involved in olfaction [21], while the mouth parts contain the pedipalps and chelicerae, which are presumed to play a role in gustatory functions through their respective sensilla [4,15]. Thus, the paired forelegs and mouth parts are likely associated with olfactory- and gustatory-related genes, respectively [5]. Due to the difficulty of collecting sufficient chemosensory tissue parts from nymph ticks because of their small size, we dissect the nymphs and consider the anterior portion that contains these chemosensory structures as chemosensory tissues, while the posterior portion, which lacks these structures, as well as the hindlegs of adult ticks, were considered non-chemosensory tissues [24]. The putative chemosensory-related gene HlonML-1 exhibited significantly higher expression levels in both 5% carbon dioxide (CO_2_) gas and ammonia solution (NH_4_OH) compared to non-exposed ticks (Figure 3B). Additionally, the relative expression level of HlonML-1 was substantially higher in the chemosensory-related tissue, the forelegs of unfed adult ticks (Figure 3C), and the anterior portion of unfed nymph ticks (Figure 3D) than in the hindlegs and posterior portion, respectively.

In addition to these peripheral tissues, the expression profile of HlonML-1 was further analyzed in several visceral tissues, including the fat body (Fb), salivary glands (Sgs), ovary (Ov), midgut (Mg), and hemolymph (Hem) of partially engorged (four days fed) adult ticks. This gene was found to be most abundant in the fat body, which was followed by the hemolymph (Figure 3E). Gene expression analysis was also conducted across different developmental stages and feeding statuses. HlonML-1 showed relatively high expression levels in adults, which was followed by nymphs. Regarding feeding status, a significant difference in expression was observed in adults with unfed adult ticks showing significantly higher levels than fed ones (Figure 3F). Since our study focused on parthenogenic *H. longicornis*, we could not assess variations across sex groups that could likely implicate whether the candidate gene’s role is related to locating a partner (male-biased) or host-seeking behavior.

### 3.4. Effect of HlonML-1 Gene Silencing on Ticks’ Response to Selected Odorants

**Figure 4:**
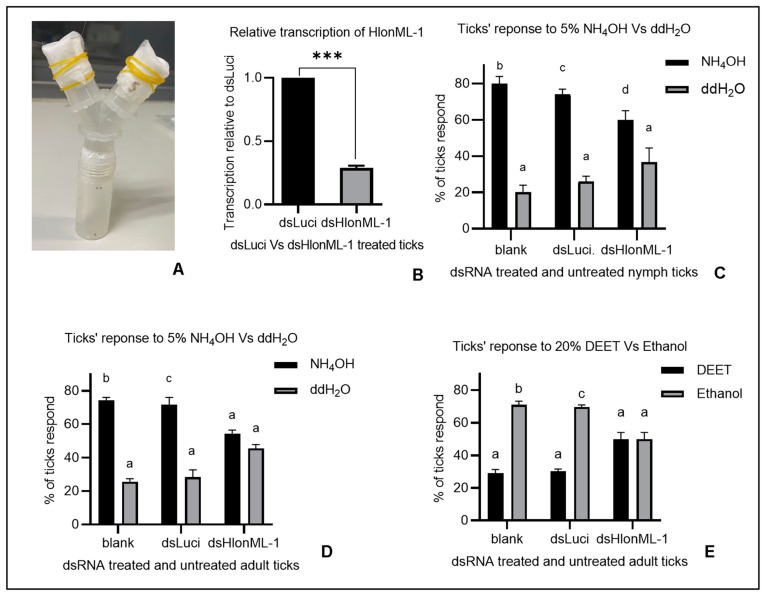
The impact of HlonML-1 gene silencing on *H. longicornis* olfactory behavioral response to selected odorants. Y-tube olfactometer apparatus (**A**). HlonML-1 gene knockdown efficiency confirmed by RT-qPCR (**B**). Percentage of ticks treated with dsRNA (against HlonML-1 and Luciferase/Luci (control)) and untreated (blank) ones respond toward 5% NH_4_OH (tick attractant) or its solvent (double-distilled water) in both nymphs (**C**) and adult ticks (**D**). Percentage of adult ticks treated with dsHlonML-1 and dsLuci and untreated ticks respond toward 20% DEET (tick repellent) or its solvent (pure ethanol) (**E**). Different letters indicate the presence of a significant difference (*p* < 0.05) while the same letter implies no significant difference. Asterisks indicate the level of statistically significant differences (*** *p* < 0.001).

A Y-tube olfactometer apparatus (Figure 4A) was used to evaluate the olfactory behavioral response of *H. longicornis* ticks to selected odorants. In this setup, volatile odorants were employed to attract or repel ticks, requiring them to move or climb while detecting these scents from a distance without direct contact. Our RNAi approach demonstrated a significant reduction in transcript levels in ticks injected with dsHlonML-1 compared to those injected with dsLuci (control) (Figure 4B). Two groups of unfed nymph and adult *H. longicornis* ticks were injected with dsRNA; one group received HlonML-1 dsRNA (treatment group: dsHlonML-1), while the other received Luciferase dsRNA (control group: dsLuci), along with a third non-injected group (test control: blank).

The percentage of ticks that responded to either odorant or solvent was then computed among those tested in the dual choice Y-tube olfactometer assay. Notably, a significant percentage of dsHlonML-1 treated nymph ticks responded to NH_4_OH compared to its solvent (ddH_2_O), similar to the control group (Figure 4C), indicating that attraction behavior was retained. However, the reduction in HlonML-1 expression did not significantly affect the percentage of dsHlonML-1-treated adult ticks that moved toward 5% NH_4_OH or its solvent, unlike the control group (Figure 4D). Unlike the control group, adult ticks treated with dsHlonML-1 showed no significant difference in their response to DEET or its solvent (pure ethanol) (Figure 4E). Unfortunately, after dsRNA injection, a relatively smaller number (about 44%) of nymphs chose either side (NH_4_OH or ddH_2_O) of the Y-tube compared to over 53% of adult ticks. The response rate was even lower (<30%) for dsRNA-injected nymph ticks tested against DEET and its solvent. Therefore, following the decision made in a previous study [49], we avoided analyzing data when less than 35% of tested ticks failed to choose either side of the Y-tube to prevent biasing the results.

The percentage of dsLuci-injected and non-injected adult ticks (control groups) demonstrated a significantly greater preference for ammonia than water and ethanol over DEET. This confirmed the intended impact of the tested odorants, which significantly triggered the ticks’ olfactory behavioral responses. In contrast, no notable differences were observed in the preferences of dsHlonML-1-injected adult ticks across either side of the arena. This suggests that the ability of adult ticks to detect the tested odorants (NH₄OH and DEET) may be impaired due to the reduction in HlonML-1 transcript levels. Conversely, dsHlonML-1-injected nymph ticks appeared to prefer the ammonia solution over water, which was similar to the response of nymph ticks in the control group. This indicates that they retained their capacity to detect the attractant compound (NH₄OH) despite the transcriptional decrease in the target gene. These findings imply that while a reduction in HlonML-1 transcript levels affects the ability of adult *H. longicornis* ticks to sense odorants such as ammonia and DEET, nymph ticks’ ability to sense ammonia remains unaffected. This may indicate its involvement in olfaction in adult *H. longicornis* ticks but may not regarding nymphs and the ammonia odor. Further validation through alternative methods, such as advanced gene editing and electrophysiological recordings along with additional odor sources (potential kairomones and pheromones), should complement these findings, particularly for tick tests at the nymph stage.

### 3.5. Effect of HlonML-1 Gene Silencing on Ticks’ Attachment Rate to a Rabbit Ear

**Figure 5:**
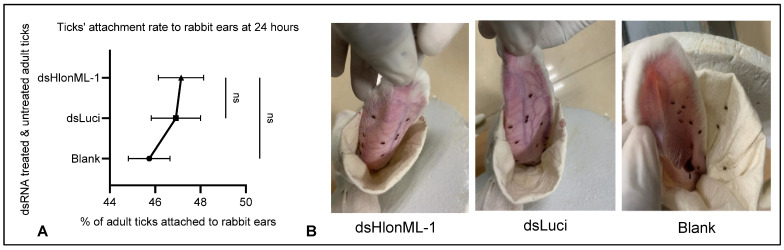
The effect of HlonML-1 gene silencing on adult *H. longicornis* ticks’ attachment rates to rabbit ears. Comparing the percentage of dsHlonML-1 treated adult ticks to dsLuciferase/Luci-treated (control) and untreated (blank) ticks attached/feeding into rabbit ears at 24 h (**A**). An illustration representing the *H. longicornis* ticks of these three groups placed into shaved rabbit ears secured with ear bags was observed after 24 h (**B**). Data are expressed as percentage mean ± SEM. The symbol “ns” indicates the absence of statistically significant differences.

The HlonML-1 gene was expressed in the mouth part of adult *H. longicornis*, but the difference was not statistically significant compared to the control tissue. Therefore, we further evaluated the potential role of this gene in gustatory chemosensation. Ticks were placed inside ear bags to allow them to come into contact with the external skin of rabbit ears, where naturally secreted kairomones are likely present. Gustatory-related receptors in the mouth parts (chelicera and pedipalps) are thought to be responsible for gustation and host differentiation [4,15]. Additionally, removing the pedipalps has been shown to affect tick attachment behavior [5], which is an initial step in feeding when ticks insert their mouth parts into the host’s skin. However, dsHlonML-1-injected adult ticks did not show any significant difference in attachment rates (measured by the insertion of the tick’s mouth parts into the rabbit ear) at 24 h compared to dsLuci-injected and non-injected control ticks (Figure 5). This indicates that the transcriptional reduction of the HlonML-1 gene does not affect the attachment rate of adult *H. longicornis* ticks to shaved rabbit ears. Given the host-specific nature of ticks, future studies should incorporate various phagostimulant compounds to further validate the role of HlonML-1 in gustation.

## 4. Discussion

A microplusin-like gene, HlonML-1, is predicted to encode a small secretory protein characterized by conserved cysteine residues and a predicted signal peptide at its N-termini. These conserved cysteine residues indicate that HlonML-1 may remain stable in extracellular environments. These features are typical of semiochemical binding proteins found in non-insect arthropods [20]. Notably, despite having relatively fewer cysteine residues, the lack of sequence similarity between HlonML-1 and the antimicrobial peptide microplusin is consistent with findings by Zhang et al. [50], who reported a cysteine-rich antimicrobial peptide that showed no identity in BLAST searches. Importantly, histidine residues that form a copper-binding site essential for the antimicrobial function of microplusin [29] are absent from the HlonML-1 protein sequence. This absence raises doubts about the antibacterial efficacy of such microplusins [28], as indicated in the Ixodes species. In addition to HlonML-1’s unique sequence, which lacks the antimicrobial basics, it appears to be evolutionarily related to existing putative chemosensory MLs identified by Renthal et al. [22]. This positions HlonML-1 as a promising candidate for chemosensory-related functions in *Haemophysalis longicornis*. We concur with the suggestions made by Josek et al. [25] and Renthal et al. [22] regarding the potential chemosensory roles of microplusin-like genes in ticks.

Unfortunately, although our anti-Gst-HlonML-1 sera successfully detected the native HlonML-1 protein in whole-body tissue extracts from unfed adult ticks (Figure 2C), it proved ineffective in detecting this protein in chemosensory tissue extracts (forelegs and mouth parts) of *H. longicornis*. This result echoes the difficulties encountered by Iovinella et al. [24] in detecting a small soluble potential chemosensory protein (Neiman pick type C2) from the forelegs of *I. ricinus* using Western blotting. However, they successfully detected it within the chemosensory sensilla located in the forelegs (Haller’s organ) and mouth parts (palp) through immunocytochemistry. The failure to detect HlonML-1 in these chemosensory structures using Western blotting may be attributed to the possibility that our mouse-generated polyclonal antibody lacks sufficient affinity to detect a potentially low-abundance native protein concentration in these specific tissues. Additionally, this issue could be related to the challenges associated with the protein extraction process from these peripheral chemosensory tissues that are hard and fibrous. Therefore, in this regard, techniques such as immunocytochemistry, which do not require protein extraction, may serve as more effective alternatives.

The biased expression of HlonML-1 in potential chemosensory tissues, particularly in the forelegs of adult *Haemophysalis longicornis*, aligns with findings from a comparative analysis of tick forelegs in *Ixodes ricinus* [24] and *I. scapularis* [25]. These studies suggest that candidate chemoreceptors that are differentially expressed in forelegs are likely to contribute to the functions of Haller’s organ. The foreleg of the tick is considered a sensory structure housing Haller’s organ, which is a unique chemosensory organ that is believed to play a crucial role in olfaction by detecting host odors and pheromones [5,21]. Furthermore, the expression level of HlonML-1 significantly increased in ticks exposed to 5% (CO_2_ and NH_4_OH) compared to unexposed ticks. These results corroborate findings from Cui et al. [23], which indicated that the expression levels of soluble olfactory-related genes in *H. longicornis* were elevated following exposure to ammonia odors. The carbon dioxide gas and ammonia solutions are known tick attractants [38,51] that activate the olfactory sensilla associated with Haller’s organ [49,52], suggesting that HlonML-1 likely plays a role in the olfactory mechanisms of *H. longicornis*. Additionally, HlonML-1 exhibits a relative abundance in adult and unfed ticks compared to their fed counterparts. This observation aligns with previous research indicating that chemosensory-related genes are downregulated in blood-fed adult females of *Dermacentor variabilis* [5]. Such changes may reflect ticks’ evolving physiological and behavioral needs, particularly the potential decline in host-seeking behavior among fed ticks, as they no longer require a blood meal [53], in which chemosensation is crucial.

In addition to its relative abundance in the chemosensory tissues of *H. longicornis*, HlonML-1 was also expressed in various visceral organs with higher levels found in the fat body and hemolymph compared to the midgut and salivary glade. This expression distribution contrasts with that of the antimicrobial peptide microplusin, which has been reported to be differentially expressed in the salivary gland and midgut of *Amblyomma aureolatum* [28]. This variation in expression sites may be related to differences in their function, suggesting that HlonML-1 might have evolved for a new role in ticks. Notably, given HlonML-1’s broad expression profile, including in non-chemosensory tissues, it may exhibit multifunctional roles similar to those observed in the insect chemosensory protein family (CSP), which are believed to have evolved for diverse biological functions beyond mere chemosensation [54,55], which is a hypothesis that warrants further investigation. A significant decrease in HlonML-1 transcription was obtained in *H. longicornis* ticks (Figure 4B). This allowed us to assess the potential impact of silencing the target gene on chemosensory-related behavioral changes in ticks. Ammonia is known to influence host-seeking behavior in ticks [12,38,51], while DEET is recognized as the most commonly used tick repellent [56]. A significant reduction in HlonML-1 transcription levels affected the detection of ammonia solution (NH_4_OH) and *N*,*N*-diethyl-3-methylbenzamide (DEET) by adult *H. longicornis,* unlike the adult ticks in the control group (Luciferase-dsRNA treated). This suggests that HlonML-1 may play a role in the olfactory chemosensation of matured *H. longicornis*.

As we further evaluated the potential role of HlonML-1 in gustation, during a 24-hour observation, less than 50% of Luciferase dsRNA-treated *H. longicornis* control ticks (Control group) were attached to rabbit ears, which is notably lower than the rates of up to 76.7% observed in *Rhipicephalus haemophysaloides* control ticks [41]. This variation in attachment rates may be due to differences between species. HlonML-1 is present in the mouth parts, which contain sensillum crucial for taste perception [4] and detecting contact and short-range cues [57], which potentially affects the ticks’ differentiating and attachment rates to their hosts’ skin. A significant reduction in its transcription did not notably influence the percentage of dsHlonML-1-treated adult *H. longicornis* ticks attached to rabbit ears during the observation period compared to the control ticks. Therefore, it can be inferred that while HlonML-1 may contribute to olfaction, it does not appear to impact gustation in adult *H. longicornis* when feeding on rabbit ears. However, assays using live hosts may provide ticks with a broader range of potential kairomones. Given the host specificity of ticks, further experiments independently testing various phagostimulant compounds could enhance our understanding of gustatory-related findings.

Unfortunately, unlike the dsRNA-untreated ticks, *H. longicornis* nymph ticks treated with dsRNA did not exhibit a distinct preference for moving toward or away from the repellent odor (DEET) or its solvent (pure ethanol), which was excluded from analysis. This observation aligns with findings that indicated over 65% of ticks failed to respond to either side of the Y-tube olfactometer apparatus [49], leading the authors to suggest that this method may not be suitable for repellency testing. The Y-tube olfactometer assay has been effectively utilized to evaluate the olfactory responses of ticks [38,47,49], including the repellency behavior of *H. longicornis* nymph ticks [36]. However, this lack of response may be due to the synergic impact of several factors, including the small size of nymphs to accommodate the volume of injection resulting in injury/discomfort as well as the deterrent nature of DEET that diffuses through the Y-tube olfactometer. Therefore, it can be suggested that when employing such gene functional experiments involving the nymph ticks’ repellency behavioral test [34,56], it may be beneficiary to adopt techniques that minimize discomfort due to injections (e.g., immersing nymphs in dsRNA solution) or use a method that does not require the ticks to move (e.g., electrophysiological recordings). Advanced methods such as gene editing at an egg stage may also provide a better alternative.

We believe that further validating the existing putative chemosensory microplusins identified in the genomic data of multiple tick species could significantly contribute to elucidating the olfactory roles of these candidate genes in ticks. We also suggest conducting additional experiments, such as protein–ligand binding assays and electrophysiological recordings incorporating various phagostimulants and odor sources. Furthermore, assessing the relative expression of the target gene in ticks from different sex groups could enhance our understanding of its specific role in the olfactory system. This research may contribute to identifying novel molecular targets that are crucial for developing olfactory-based tick control strategies.

## 5. Conclusions

Microplusin, known for its antimicrobial role, has recently been proposed to have a potential chemosensory function in ticks. Our research focused on a microplusin-like gene in *Haemophysalis longicornis*, which was designated as HlonML-1. This gene exhibits a unique sequence that suggests a role in olfaction, which is critical for host-seeking behavior, especially as it is preferentially expressed in chemosensory tissues like the forelegs. Silencing HlonML-1 impairs the odor-detection capabilities of adult ticks, indicating its significant role in their olfactory chemosensation. In addition to investigating other putative chemosensory microplusins in ticks, future relevant experiments, including protein–ligand binding assays, could further elucidate HlonML-1’s specific role in the olfactory process.

## Data Availability

The original contributions presented in the study are included in the article/Supplementary Material; further inquiries can be directed to the corresponding author/s.

## Supplementary Materials

The following supporting information can be downloaded at: https://www.mdpi.com/article/10.3390/microorganisms12112269/s1, Table S1. Sequences for the microplusin-like (ML) candidates; Table S2. The primer sequences lists, and Figure S1. Original images for HlonML-1 identifications in gels/blots.
